# The influence of maternal health education on the place of delivery in conflict settings of Darfur, Sudan

**DOI:** 10.1186/s13031-015-0057-2

**Published:** 2015-10-05

**Authors:** Izzeldin F. Adam

**Affiliations:** Department of International Health and Medicine, Division of Public Health, Graduate School of Tokyo Medical and Dental University, Yushima 1-5-45, Bunkyo-ku, Tokyo 113-8519 Japan; Department of Epidemiology, Faculty of Public and Environmental Health, University of Khartoum, Khartoum, Sudan

**Keywords:** Home visits, Maternal education, Home-based delivery, Conflict-affected settings, Darfur-Sudan

## Abstract

**Background:**

Armed conflict and socio-demographic characteristics of internally displaced persons (IDPs) are very important factors that influence the provision of reproductive health (RH) in humanitarian settings. Maternal health education plays a crucial role to overcome the barriers of RH care, reduce home births conducted by traditional birth attendants (TBAs), and improve increasing births in a health facility. The objectives of this study were to (1) determine the association between the place of delivery and home visits for maternal health education and (2) describe the socio-demographic characteristics of women who gave birth during the last two years.

**Methods:**

A cross-sectional study among married women aged (15–49 years old) in IDP camps. All women were subjected to intensive maternal health education at their homes for 3 years prior to the survey. A sample of 640 women who gave birth during the last two years was randomly selected.

**Results:**

Among all women investigated, 36.9 % (95 % CI: 33.1, 40.8) reported a home-based delivery, while 63.1 % (95 % CI: 59.2, 66.9) reported a facility-based delivery. Receiving visits for maternal health education at home was associated with an estimated 43.0 % reduction in odds of giving birth at home, compared to not receiving home visits (adjusted odds ratio [ aOR] 0.57; 95 % CI: 0.35, 0.93). The level of women’s education and camp of residence were important predictors for home birth.

**Conclusion:**

Maternal health education at home was associated with a reduction in home-based delivery performed by TBAs in the conflict-affected setting of Darfur. Our study proposes that when facility-based delivery is made available in camp’s clinics, and the targeted women educated at home to refrain from home-based delivery, they will choose to undergo facility-based delivery.

## Background

In conflict-affected settings maternal health may be worsened by displacement, limited resources, inaccessibility of RH care, and destruction of health facilities. Despite the global initiative of safe motherhood, launched by the WHO in 1987, home-based delivery performed by TBAs in unhygienic environments is still common in resource-poor settings [1–3].

Based on scientific evidence, poor socioeconomic status, residence in resource-poor settings in rural areas, low level of education, conservative culture, harmful traditional practices, and low awareness of skilled birth attendance in a health care facility are strongly associated with poor maternal health indicators, especially home-based delivery [4–8].

In many cases in developing countries, maternal deaths among women in neglected communities are associated with home delivery [9]. These deaths are avoidable by creating awareness of safe motherhood and accessing facility-based delivery performed by skilled birth attendants [5, 9, 10].

In Sudan, almost 60 % of rural areas do not have access to even basic midwifery services. Midwives and TBAs are the main trusted caregivers in their remote communities [11, 12]. Countrywide, only 66 % of national health facilities can provide basic emergency obstetric and newborn care (EmONC) and only 46 % can provide comprehensive EmONC [11], while only 21 % of deliveries are being conducted in health facilities [13].

The package of basic EmONC which can be provided with skilled staff in health centers, includes the capabilities for: administering antibiotics, uterotonic drugs (oxytocin) and anticonvulsants (magnesium sulphate); manual removal of the placenta; removal of retained products following miscarriage or abortion; assisted vaginal delivery, preferably with vacuum extractor; and basic neonatal resuscitation care. However, the comprehensive EmONC delivered in hospitals, includes all the basic functions plus capabilities for: performing caesarean sections; safe blood transfusion; and provision of care to sick and low-birth weight newborns including resuscitation [14].

In the Darfur region of Sudan, conflict between the central government and armed rebels fighting for redistribution of wealth and power has resulted in a massive displacement [15]. According to United Nations estimates, more than 2 million people fled their homes and around 200,000 people have died since the war started in 2003 [16]. High rates of mortality have been estimated in both the pre-displacement and post-displacement periods [17, 18]. As reported in the Sudan Household Health Survey (SHHS) 2006, a very high maternal mortality ratio (MMR) of 1,056 deaths/100,000 Live Births (LB) has been reported in West Darfur [19]. In this marginalized region, the health care system is very poor and lacks even the basic health services. The access to maternal health care is very limited and endangered by the insecure situation, and women in Darfur are more likely to die in pregnancy, delivery, and the postpartum period [11]. The access to primary health care (PHC) facilities varies from 1: 20,779 people in South Darfur to 1:3,039 people in the Northern State; while the national average is 1:6,816 people. Furthermore, there are only 0.2 hospitals and 14 beds per 100,000 in South Darfur compared to 5.2 hospitals and 246 hospital beds per 100,000 people in the Northern state [20].

Sudan severely suffers from shortage of medical personnel providing EmONC, especially at rural areas of Darfur. Nearly two-third of health workforce in the country are living and working in urban settings [20]. According to SHHS 2010 [13], village midwives have assisted one-half of all births (49.3 %), while figures of deliveries with the assistance of a trained attendant being lowest in West Darfur State (33.4 %) and highest in Northern State (96.7 %). Nationwide, the density of medical doctors, nurses and midwives is only 1.23 per 1000 population. This makes the country within the critical shortage zone according to the WHO benchmark of 2.28 health care professionals per 1000 population [20].

There is an increasingly visible gap between access to maternal health services in Darfur and other regions of the Sudan. The national statistics of the Sudan Household Health Survey 2006 show that a large proportion of women in Darfur had no access to delivery in health facilities and other maternal care services [19].

Based on scientific evidence, most maternal deaths can be prevented if timely and adequate maternal care is provided [21–23]. In response to the emergency situation in Darfur, Save the Children launched an emergency RH program in West Darfur in 2005 [24]. West Darfur state is located in the far West of the Sudan with international borders of 760 km with Chad and Central Africa. Our study took place in three IDP camps namely; Krending, Krenik, and Habillah. These camps are located 25 km east, 46 km east, and 54 km south, respectively, of West Darfur’s capital city, Geneina. The camps were populated with 88,984 IDPs descended from various tribes of different dialects who fled from different parts of the marginalized communities in the region. The proportions of population were 21,990, 40,403, and 26,591 IDPs per camp, respectively. The program was aimed at reducing maternal mortality and improving women’s health by providing a package of RH services. The package included: Basic EmONC; antenatal care; family planning services; immunization; nutrition; hygiene promotion; STIs and HIV/AIDS prevention. The services were available free of charge according to guidelines of Minimum Initial Service Package (MISP) [25]. In each camp, one primary health care clinic (PHCC) with two peripheral units was installed. Three medical doctors, one medical assistant, nurses, and midwives were recruited.

Women in rural areas of the Darfur region have a strong attachment to the traditional maternal practices in home-based delivery performed by TBAs such as carrying out the delivery on the ground or a local mat and using unsterilized equipment to cut the umbilical cord [26]. Therefore, the utilization of institutional delivery was very low. To overcome these barriers, Save the Children and West Darfur Ministry of Health created a complementary community-based approach of maternal health education through home visits that aimed to decrease home-based delivery and create demand for facility-based delivery. Three main PHCC were installed in 2005 in IDP camps Krending, Krenik and Habillah. To ease access, the clinics were constructed within reasonable distance (one clinic per 10,000 persons). For each camp, two additional peripheral outpatient clinics were established to support the main PHCC and serve as a first contact point. Each PHCC was equipped to provide antenatal care, basic EmONC, family planning, and postpartum care. Moreover, for each camp, an ambulance was provided for the referral of prolonged and obstructed labor to a referral facility in West Darfur. The referral facility was the only available comprehensive EmONC facility for our three camps [24]. Sixty-two female maternal health workers (MHWs) were selected and trained to conduct maternal health education at homes of internally displaced women. Each woman was targeted to receive at least one monthly visit for maternal health education at her home. The interpersonal community communication was continued side-by-side with reproductive health care services in clinics for three years in all camps of operation.

In addition, little is known about the provision of facility-based delivery by NGOs and the socio-demographic determinates of place of delivery in conflict affected settings. We conducted a cross-sectional survey to (1) determine the association between place of delivery and home visits for maternal health education, and (2) describe the socio-demographic characteristics of women with delivery.

## Methods

### Design

A cross-sectional study by closed-ended; interviewer-administered questionnaire was conducted in April 2009 in IDP’s camps in the West Darfur region of Sudan among married women of child-bearing age (15–49). The evaluation tools were selected and adapted from the Reproductive Health Response in Conflict (RHRC), Consortium’s Health Needs Assessment, Field Tools and the Monitoring and Evaluation Toolkit [25, 27]. Data were collected on participants’ socio-demographic characteristics, receiving home visits for maternal health education, and place of delivery. In this study, our primary outcome was place of delivery (home-based delivery conducted by TBAs vs. facility-based delivery). The main independent variables were the socio-demographic characteristics of participants, i.e. age, education, employment, and camp of residence. The main exposure was home visits for maternal health education. The main outcome variable was the place of delivery. The questionnaire was translated into Arabic and back translated into English to ensure accuracy. Completed questionnaires were checked by field supervisors. To ensure data accuracy and satisfy cultural aspects, only female local interviewers who were residents of the same camps but hadn’t participated in the intervention were recruited. All the interviewers received intensive training for 5 days prior to the survey. The interviews generally took place at the respondent’s household (shelter), where interviewers ensured auditory privacy.

### Settings

Female maternal health workers were selected from local communities after they were recommended by tribal leaders, and trained intensively for three weeks in reproductive health communication. Our training focused on the skills of establishing active communication with respondents such as active listening, talking technique, building trust, facilitating decision making, linking respondents with resources of support in health care facilities. The basic information about the components of RH in crisis were highlighted in the training with special focus on danger signs during pregnancy, antenatal care, maternal and newborn care, risk of traditional birthing methods, help-seeking behaviors, access to maternal health support, etc. MHWs were divided into small teams to cover all women monthly at their homes. To achieve this, each MHW was assigned to work for 3 days per week and visit 20 women per day.

### Intervention

Overall, 17,796 married women of child-bearing age (15–49 years old) were targeted in March 2006 by home visits for maternal health education (door-to-door). During this intervention, interactive discussions between MHWs and women were conducted in a private environment at home. Materials of information, education, and communication (IEC) such as posters, flipcharts, and brochures were used for demonstrations. Simple and clear messages were presented to highlight the risk of home-based delivery and to value the importance of facility-based delivery. Frequent visits were undertaken for monitoring of knowledge gained, observing behavioral change process, and supporting women to make a decision on their own health status and start seeking delivery in health care facilities.

In this intervention, our theoretical framework was based on personal, enabling, and need characteristics of participants. In our home visits for maternal health education, we individualized health messages based on personal characteristics of women (e.g. age, level of education, employment, and camp of residence). The enabling characteristics emphasized on maternal health services (i.e. ease of access to camp clinic or referral facility using ambulance and availability of free basic EmONC). The need characteristics of our intervention emphasized the expected risk of home-based delivery and the perceived benefit from facility-based delivery [7, 8, 28].

### Subjects and sampling procedures

Our eligibility criteria was set for a married woman of child-bearing age (15–49 years old) who joined the camp during the last three years prior to the survey (during the intervention), had experienced pregnancy, was sexually active, and had an opportunity to benefit from maternal health services at the camp’s clinic and to receive maternal health education at home. To determine a desired sample size, we used a precision-based approach with design effect of 1.65 and a 95 % confidence interval. The design effect was randomly generated using a random number function between 1.5 to 2.0 on a scientific calculator to correct the estimated sampling variance for efficient sample size in a cluster survey. This design has become a standard method of assessing health and nutrition status in emergency situations, where populations are large and no sampling frame exists to permit simple or systematic random sampling. The minimum sample size of households to be visited to interview eligible women was set at 633, then, rounded to 640 women. The total estimated number of married women of child-bearing age in the three camps was 17,796.

**Fig. 1:**
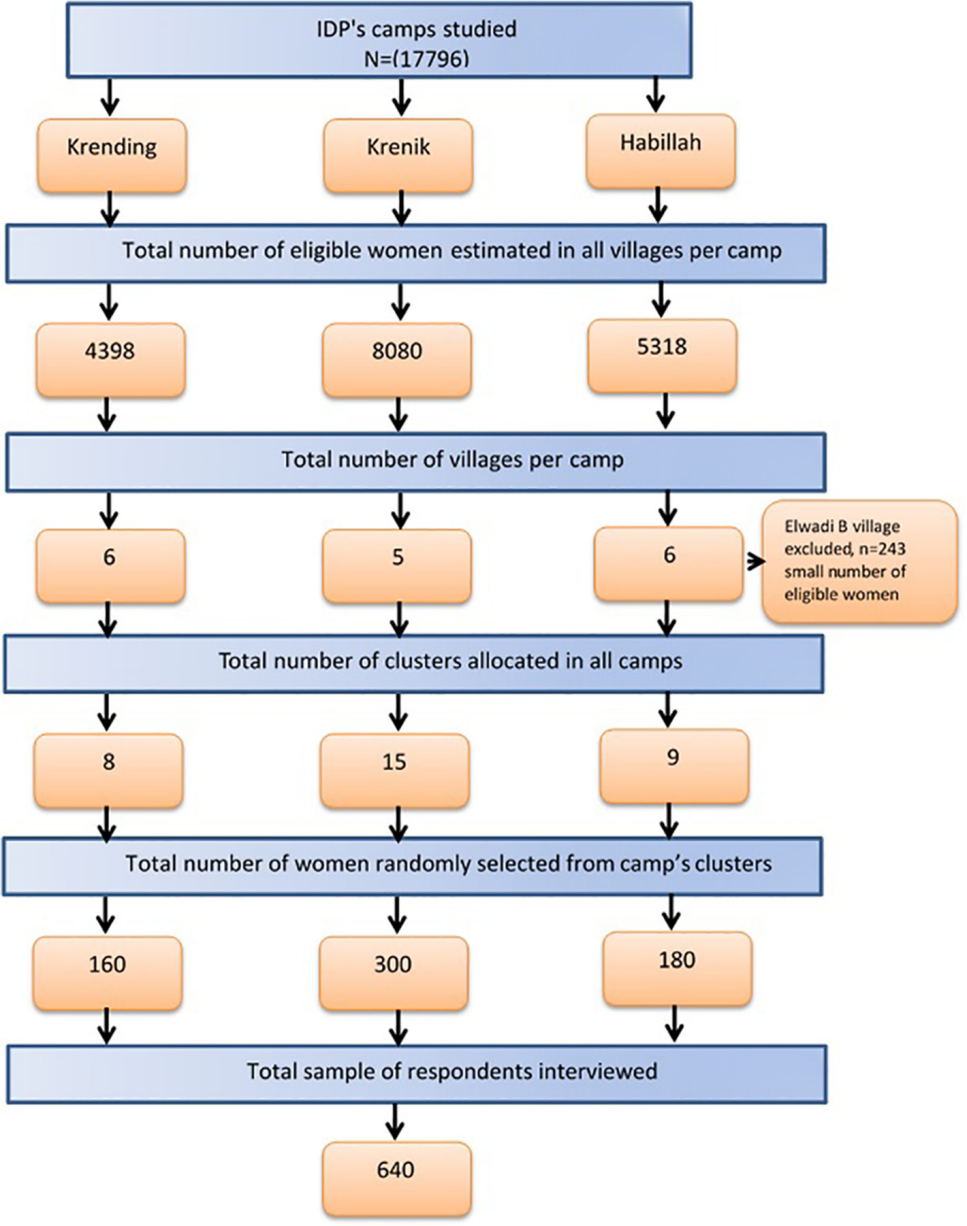
Study flow chart of sample selection for married women of child bearing age (15–49 years old) in internally displaced person’s camps in 2009, Darfur, Sudan

Thirty-two clusters were required by using a cluster size of 20 households (640 / 20 = 32). In the first stage, clusters were allocated using probability proportional to the size of women populations in each camp. The total number of women was divided by the total number of clusters, the interval was estimated at 556 (17,796/32 = 556). To determine the location of clusters within camps, local maps were drawn to show roads and population distribution. Each camp was delineated into its small population units (villages) which were established according to the arrival of IDPs to a certain camp. Our three camps included 17 villages (8 in Krending; 15 in Krenik; 9 in Habillah). All villages were randomly listed in order; the number of eligible women was estimated for each village; then, the cumulative numbers were ordered to determine the number of cluster/s in each village. The location of the first cluster was determined using a random number between one and the sampling interval using a scientific calculator, and then other clusters were selected by successive additions of the sampling interval. In our context, the cluster was a homogeneous socio-cultural population unit within the IDP’s camp that contained at least 556 married women of child-bearing age (15–49). We defined the household as a domestic unit consisting of cohabiting family members, including husband, one or more wives, children, and grandparents (if any). In the second stage, in each cluster 20 households were randomly selected to interview eligible women. In the case of more than one eligible woman in a single household, only one woman was randomly selected. If there were no women in the selected households or they were absents, the nearest household was selected, but there was no refusal case. All clusters were reachable and there was no replacement, but our formula did not allocate any cluster in Elwadi B village, which was the smaller village with only 243 eligible women (Fig. 1).

### Analysis

Pearson’s chi-square was used to assess differences across socio-demographic characteristics and receiving maternal health education for all women and by place of delivery. The proportion of women who reported reasons for home delivery attended by TBAs was calculated for all women and by camp of residence.

We used logistic regression models to control the potential confounders. Univariate and multivariate analyses were conducted for the place of delivery (delivery at home vs. delivery in health care facility) to measure the crude and adjusted odds ratios (OR). Based on previous studies, the independent variables of socio-demographic characteristics were considered as potential confounders for receiving maternal health education at home. We generated a univariate analysis between each independent variable and home-based delivery. Any variable showing a tendency of association with outcome was included in the multivariate analysis (*P* value cut-off point of 0.25). We used a backward selection procedure to generate a parsimonious final model. The model adjusted for age, education, employment, camp of residence, and receiving maternal health education at home (i.e. significant at *P* < 0.05). The procedures for complex samples logistic regression analyses on a binary dependent variable for subpopulations were drawn .We optionally requested the analyses to fit our model and control the display of statistics that measure the overall model performance and estimate confidence intervals. The covariates were specified in the main dialog box; then, a custom category was set as the reference category. All data were entered into IBM SPSS statistics version 22.0 for analysis.

### Ethical considerations

The study design and data collection instruments were reviewed and approved by the institutional review board of the Faculty of Public Health, University of Khartoum. Moreover, the study proposal was approved by the Federal Ministry of Health, Sudan; and the Humanitarian Aid Commission. All the participants were informed about the objective and procedures of the study, then, verbal informed consents were obtained as a mandatory requirement prior to participation in the interview.

## Results

Table 1 represents data for place of delivery, socio-demographic characteristics, and receiving home visits for maternal health education among women in IDP camps in Darfur. We analyzed data for 640 women, the overall response rate was hundred percent, of whom 36.9 % (95 % CI: 33.1, 40.8) reported home-based delivery, while 63.1 % (95 % CI: 59.2, 66.9) reported facility-based delivery. Among the socio-demographic characteristics, only education and camp of residence were significantly associated with place of delivery (both *P* value < .001), while age and employment were not significantly associated with place of delivery. Our main exposure of home visit for maternal health education was statistically significant with place of delivery. The dominant age group of 31–40 years old reported the highest percentage of home delivery of 35.2 % (95 % CI: 29.1, 41.3), however, the younger and older age groups showed smaller proportions of home delivery. More than half of the women (59.1 %; 95 % CI: 55.5, 62.7) did not receive formal education and/or were illiterate, of whom 73.7 % (95 % CI: 68.1, 79.4) reported home delivery. More than two thirds of women (67.0 %; 95 % CI: 63.3, 70.5) were employed in simple jobs, whereas of them 62.7 % (95 % CI: 56.5, 68.9) reported home delivery. Nearly half of the women (46.9 %; 95 % CI: 43.1, 50.8) were located in Krenik, of whom 37.3 % (95 % CI: 31.1, 43.5) reported home delivery. The vast majority of women (86.7 %; 95 % CI: 84.2, 89.4) received home visits for maternal health education, of whom more than three quarters underwent facility-based delivery.

**Table 1:** Socio-demographic characteristics and receiving maternal health education at home by place of delivery among women in internally displaced person’s camps in 2009, Darfur, Sudan

Basic characteristics		All women (*N* = 640)		*P* value	Place of delivery			
					Home-based delivery *N* = 236; 36.9 % (95 % CI: 33.1, 40.8)		Facility-based delivery *N* = 404; 63.1 % (95 % CI: 59.2, 66.9)	
		*n*	% (95 % CI)		*n*	% (95 % CI)	*n*	% (95 % CI)
Socio-demographic characteristics								
Age								
	<20 years old	123	19.2 (16.6, 22.2)	.060	50	21.2 (16.0, 26.4)	73	18.1 (14.3, 21.8)
	20-30 years old	225	35.2 (31.4, 38.7)		82	34.7 (28.6, 40.8)	143	35.4 (30.7, 40.1)
	31-40 years old	253	39.5 (35.8, 43.1)		83	35.2 (29.1, 41.3)	170	42.1 (37.3, 46.9)
	>40 years old	39	6.1 (4.2, 7.8)		21	8.9 (5.3, 12.5)	18	4.5 (2.4, 6.5)
Education								
	No formal education/none	378	59.1 (55.5, 62.7)	<.001	174	73.7 (68.1, 79.4)	204	50.5 (45.6, 55.4)
	Basic school	201	31.4 (28.0, 34.8)		57	24.2 (18.7, 29.6)	144	35.6 (31.0, 40.3)
	≥ Secondary	61	9.5 (7.3, 11.9)		5	2.1 (0.3, 4.0)	56	13.9 (10.5, 17.2)
Currently employed								
	Yes	429	67.0 (63.3, 70.5)	.076	148	62.7 (56.5, 68.9)	281	69.6 (65.1, 74.1)
	No	211	33.0 (29.5, 36.7)		88	37.3 (31.1, 43.5)	123	30.4 (25.9, 34.9)
Current camp of residence								
	Krending	160	25.0 (21.9, 28.6)	<.001	84	35.6 (29.5, 41.7)	76	18.8 (15.0, 22.6)
	Krenik	300	46.9 (43.1, 50.8)		88	37.3 (31.1, 43.5)	212	52.5 (47.6, 57.4)
	Habillah	180	28.1 (24.8, 31.6)		64	27.1 (21.4, 32.8)	116	28.7 (24.3, 33.1)
Received maternal health education at home								
	Yes	555	86.7 (84.2, 89.4)	.005	193	81.8 (76.8, 86.7)	362	89.6 (86.6, 92.6)
	No	85	13.3 (10.6, 15.8)		43	18.2 (13.3, 23.2)	42	10.4 (7.4, 13.4)

*P* value from x^2^ for categorical variables

Table 2 shows the reasons behind home-based delivery among women in internally displaced person camps. Women who had preferred home-based delivery even though facility-based delivery was available free of charge attributed their practices to reasons of: traditional customs, grandmother’s advice, husband’s interest, fear of doctors, to avoid surgical intervention, and not having enough time to undergo facility-based delivery (quick delivery), the proportions were 17.8 %; ( 95 % CI: 15.0, 20.8), 3.9 %; ( 95 % CI: 2.5, 5.3), 3.0 %; ( 95 % CI: 1.6, 4.4), 5.9 %; (95 % CI: 4.1, 7.7), 9.8 %; (95 % CI: 7.5, 12.2), and 4.5 %; (95 % CI: 2.8, 6.1), respectively.

**Table 2:** Reasons behind home-based delivery among women in internally displaced person’s camps 2009, Darfur, Sudan

Reason behind home delivery		All women (*n* = 640)	
		*n*	% (95 % CI)
Traditional customs			
	Yes	114	17.8 (15.0, 20.8)
	No	526	82.2 (79.2, 85.0)
Grandmother’s advice			
	Yes	25	3.9 (2.5, 5.3)
	No	615	96.1 (94.7, 97.5)
Husband’s interest			
	Yes	19	3.0 (1.6, 4.4)
	No	621	97.0 (95.6, 98.4)
Afraid of doctors			
	Yes	38	5.9 (4.1, 7.7)
	No	602	94.1 (92.3, 95.9)
Avoid surgical intervention			
	Yes	63	9.8 (7.5, 12.2)
	No	577	90.2 (87.8, 92.5)
Did not have time (quick delivery)			
	Yes	29	4.5 (2.8, 6.1)
	No	611	95.5 (93.9, 97.2)

*P* value from x^2^ for categorical variables

Table 3 represents the association between socio-demographics, receiving maternal health education, and home-based delivery among women in IDP camps. Across socio-demographic characteristics, there were significantly lower odds of home-based delivery among women aged 31–40 years compared to those aged <20 years (aOR 0.56; 95 % CI: 0.34, 0.91). Women who reported a basic and at least secondary education levels were less likely to undergo home delivery compared to women who reported informal or no education (aOR 0.44; 95 % CI: 0.30, 0.64) and (aOR 0.11; 95 % CI: 0.04, 0.28), respectively. Camp of residence was an important predictor of home delivery, women who were located in Krenik and Habillah had lower odds of home birth delivery than those who were located in Krending (aOR 0.35; 95 % CI: 0.22, 0.54) and (aOR 0.49; 95 % CI: 0.31, 0.79), respectively. Receiving home visits for maternal health education was associated with an estimated 43.0 % reduction in odds of giving birth at home (aOR 0.57; 95 % CI: 0.35, 0.93), compared to not receiving home visits.

**Table 3:** Association between socio-demographic, receiving maternal health education at home, and home based delivery among women in internally displaced person’s camps in 2009, Darfur, Sudan^a^

Predicting factors		Number	Home-based delivery^b^		
			%	OR (95 % CI)	aOR (95 % CI)
Socio- demographic characteristics					
Age					
	<20 years	50	40.7	Reference	Reference
	20-30 years	82	36.4	0.84 (0.53, 1.31)	0.62 (0.38, 1.02)
	31-40 years	83	32.8	0.71 (0.46, 1.11)	0.56 (0.34, 0.91)*
	>40 years	21	53.8	1.70 (0.82, 3.52)	1.38 (0.63, 3.04)
Education					
	Informal /none	174	46.0	Reference	Reference
	Basic education	57	28.4	0.46 (0.32, 0.67)***	0.44 (0.30, 0.64)***
	≥ Secondary	5	8.2	0.10 (0.04, 0.27)***	0.11 (0.04, 0.28)***
Employment					
	Yes	148	34.5	0.74 (0.52,1.03)	1.25 (0.85, 1.85)
	No	88	41.7	Reference	Reference
Camp of residence					
	Krending	84	52.5	Reference	Reference
	Krenik	88	41.7	0.38 (0.25, 0.56)***	0.35 (0.22, 0.54)***
	Habillah	64	35.6	0.50 (0.32, 0.77)**	0.49 (0.31, 0.79)**
Received maternal health education at home					
	Yes	193	34.8	0.52 (0.33, 0.82)**	0.57 (0.35, 0.93)*
	No	43	50.6	Reference	Reference

*P < .05; **P < .01; ***P < .001

*OR* odds ratio, *aOR* adjusted odds ratio, *CI* confidence interval

^a^The model controlled for all other variables in the table

^b^delivery at home vs. delivery in health facility

## Discussion

In this study, our primary hypothesis was to (1) determine the association between the place of delivery and home visits for maternal health education and (2) describe the socio-demographic characteristics of women who gave birth during the last two years. Among all deliveries conducted in our project sites, 236 (36.9 %; 95 % CI: 33.1, 40.8) of internally displaced women reported a home delivery, while 404 (63.1 %; 95 % CI: 59.2, 66.9) reported facility-based delivery for their pregnancy during the last two years. Compared to the national situation reported in the Sudan Household Health Survey second round 2010, only 21 % of women underwent facility-based deliveries [13]. Data from SHHS 2006 conducted prior to our intervention shows that facility-based delivery was only 7.8 % in West Darfur. According to another study conducted in a resource-poor setting in Nigeria [29], where the culture of home-based delivery is common among African tribes, 49.0 % of women delivered at home, whereas 51.0 % delivered at the hospital, where 50.8 % of deliveries were supervised by TBAs. Women in our study justified their preference to home delivery by, among other reasons, traditional customs, grandmother’s advice, husband’s interest, fear of doctors, avoidance of surgical intervention, and contracting the delivery over a short time. Many traditional, cultural, demographic, and socio-economic factors can be considered to explain home delivery [5, 21, 29–36].

Home visit for maternal health education was independently associated with lower odds of home delivery when adjusting socio-demographic factors. Home is an appropriate place for mothers to receive maternal advice based on their own individual characteristics and social environment [30, 32, 37]. Women who got an opportunity to discuss their maternal health concerns in a private environment at home were more likely to change their attitude towards delivery in health care facilities. Consistent with other studies conducted in Tanzania, Ethiopia, and Pakistan, the tremendous efforts of community health workers to raise maternal health awareness were associated with extraordinary outcomes toward promoting maternal health, including the utilization of institutional delivery [33, 38–40]. Although we implemented the same plan in all camps to provide maternal health education at homes of women, some women did not receive our message. It is possible that women were absent during our home visits; they did not have an interest in participating in maternal education sessions; or the time of visits was not suitable for them. However, there was no statistically significant difference between camp of residence and home visits for maternal health education (unpublished finding).

Among the socio-demographic characteristics, age category of 31–40 years old, level of education, and camp of residence were associated with significantly lower odds of home birth delivery. A research finding from Zimbabwe [41] reported that age was a significant determinant of place of delivery. We found that camp of residence was a very important predictor of home delivery, women who were located in Krenik and Habillah had lower odds of home birth delivery than those located in Krending. A study in the neighboring country of Ethiopia [28] indicated that the place of residence was a very important predictor of place of delivery. Women in our camps of intervention were not a homogenous community because they fled from different areas of Darfur. The villages of origin of IDPs had huge discrepancies in terms of traditions, cultures, and socio-economic characteristics; this can explain differences in their utilization of facility-based delivery. Consistent with other finding, women of a certain ethnic group in Africa were less likely to deliver at health facilities than others [42]. In addition, IDPs in Krenik and Habillah were mixed with host communities in these two areas which had some aspect of urban life, while the residents of Krending were pure IDPs. Although IDPs living in the same camp, we found the socio-demographic characteristics of the place from which they were displaced can affect their response to the same health service provided by international humanitarian organizations. Consistent with our results, many studies conducted in developing countries have shown that education is the most important determinant of home delivery [7, 8, 43, 44]. Education was recognized substantially as an inclusive measurement that influences the socio-economic position of women, [4] and is more likely to raise a mother’s awareness about the risk of home delivery, build confidence, and develop their autonomy to make wise decisions about their own choices. However, the low level of education in low-resource settings may be an indicator of lack of knowledge concerning the possible risk of traditional birth, not seeking delivery in health facilities, and a boycott of delivery in health facilities.

Our study design could not establish a causal relationship between home visits for maternal health education and place of delivery. However, such intervention with randomization allocating participants into experimental and control groups could assist in proving the causal inference. The emergency setting accompanied with insecure situation, logistical barriers, and continuous population movement. Moreover, the right of all displaced women to benefit from our maternal health services didn’t allow us to keep some women in a control group without maternal health services in a randomized control trial design. Due to the demanding situation caused by displacement, the data was self-reported and there was no verification documentation presented during the interviews. Further, the effect of the random selection of a design effect is not a standard means of calculating the appropriate design effect. Although we did not have baseline information about facility-based delivery prior to the intervention, facility-based delivery was not available for women in their villages of origin before the crisis. Our intervention was the only available program in those camp, such that we believe that our intervention was the force behind this progress.

We controlled the possible confounders related to socio-demographic and health characteristics by applying multivariate models. We employed very high quality interviewers who were educated; properly trained; oriented on socio-cultural aspects of the area; and spoke the local dialects of IDPs. Beside the personal characteristics of interviewers, the active communication with respondents, the field monitoring for data collection, and immediate clearance resulted in high response rates and there were no missing data. The results of this study can be generalized to other conflict-affected settings with the same socio-demographic characteristics, despite the challenges of the emergency settings. We encourage other researchers to conduct comparable studies that can provide evidence-based results and guide prospective humanitarian interventions in utilization of reproductive health in conflict affected-settings.

## Conclusion

In conclusion, our recent paper documents that, irrespective of contribution of explanatory socio-demographic characteristics, receiving home visits for maternal health education was associated with significant reduction of home-based delivery. Our study proposes that when facility-based delivery is made available in camp’s clinics in conflict affected-settings, and targeted women are educated at home to refrain from home-based delivery, they will choose to undergo facility-based delivery.
